# Implementation of a Long Short-Term Memory Neural Network-Based Algorithm for Dynamic Obstacle Avoidance

**DOI:** 10.3390/s24103004

**Published:** 2024-05-09

**Authors:** Esmeralda Mulás-Tejeda, Alfonso Gómez-Espinosa, Jesús Arturo Escobedo Cabello, Jose Antonio Cantoral-Ceballos, Alejandra Molina-Leal

**Affiliations:** Tecnologico de Monterrey, Escuela de Ingeniería y Ciencias, Av. Epigmenio González 500, Fracc. San Pablo, Querétaro 76130, Mexico; mtesme@gmail.com (E.M.-T.); arturo.escobedo@tec.mx (J.A.E.C.); joseantonio.cantoral@tec.mx (J.A.C.-C.); amolina@tec.mx (A.M.-L.)

**Keywords:** mobile robot, obstacle avoidance, dynamic environment, neural networks, artificial intelligence

## Abstract

Autonomous mobile robots are essential to the industry, and human–robot interactions are becoming more common nowadays. These interactions require that the robots navigate scenarios with static and dynamic obstacles in a safely manner, avoiding collisions. This paper presents a physical implementation of a method for dynamic obstacle avoidance using a long short-term memory (LSTM) neural network that obtains information from the mobile robot’s LiDAR for it to be capable of navigating through scenarios with static and dynamic obstacles while avoiding collisions and reaching its goal. The model is implemented using a TurtleBot3 mobile robot within an OptiTrack motion capture (MoCap) system for obtaining its position at any given time. The user operates the robot through these scenarios, recording its LiDAR readings, target point, position inside the MoCap system, and its linear and angular velocities, all of which serve as the input for the LSTM network. The model is trained on data from multiple user-operated trajectories across five different scenarios, outputting the linear and angular velocities for the mobile robot. Physical experiments prove that the model is successful in allowing the mobile robot to reach the target point in each scenario while avoiding the dynamic obstacle, with a validation accuracy of 98.02%.

## 1. Introduction

Autonomous robots are machines equipped with sensors and actuators to collect data from their environment and leverage their knowledge of their world to navigate safely and purposefully, without direct human control [1]. Because of their ability to perform tasks precisely and efficiently, autonomous mobile robots are essential in industrial applications as well as in other areas, such as medicine, entertainment, education, space, mining, military, rescuing, and agriculture [2]. Human–robot interactions in these scenarios are becoming more common, and they require robots to plan their trajectories safely, in an environment with both static and dynamic obstacles, a problem that has been widely researched.

While numerous authors have presented works regarding static obstacle avoidance, few have presented algorithms specific for dynamic obstacle avoidance. Some of the solutions presented come from artificial intelligence (AI) models. Machine learning (ML), a sub-field of AI, improves systems’ performance by learning from experience through computational methods [3]. According to Ehlert [1], several ML techniques, such as reinforcement learning, neural networks, and genetic algorithms, can be applied to autonomous robots to improve their performance. Machine learning can help the robot’s trajectory planning since it allows for the robot to calculate its optimal trajectory, avoiding obstacles [4].

Bakdi et al. [5] proposed an offline path-planning strategy using a genetic algorithm, piecewise cubic Hermite interpolating polynomial, and an adaptive fuzzy-logic controller for keeping track of the robot on the desired path. This implementation proved to be efficient in a static environment, allowing the robot to plan its path and execute it without collisions. Orozco-Rosas et al. [6] developed an approach combining a genetic algorithm with membrane computing and the artificial potential field (APF) method, creating the membrane evolutionary artificial potential field (memEAPF) approach. This implementation was efficient for both path planning and execution time whilst enhanced by the genetic algorithm, which proved to be more efficient than other APF strategies in both static and dynamic simulated environments. Chang et al. [7] proposed an improved dynamic window approach (DWA) path-planning strategy based on Q-learning, a reinforcement learning algorithm. By modifying and extending the original evaluation functions of the DWA and combining it with Q-learning, they obtained higher navigation efficiency in unknown static and dynamic environments. However, dynamic obstacles were not used in the training process, which could lead to less efficiency in more complex dynamic environments. Yang et al. [8] presented a path-planning hybrid method that used improved Ant Colony Optimization (ACO) for global path planning and an improved DWA for local obstacle avoidance. The method was tested in simulated unknown dynamic environments, and results showed that the algorithm improved the robot’s navigation and dynamic obstacle avoidance capability. Wang et al. [9] proposed an improved version of the Velocity Obstacle Method (VOM) for obstacle avoidance in Unmanned Surface Vehicles (USVs), which redefined the conventional obstacle geometric model set by further analyzing the obstacles. Simulation results showed that the model could achieve more accurate obstacle avoidance action. Azizi et al. [10] developed an online obstacle avoidance and motion planning algorithm for an omnidirectional mobile robot (OMR) by combining a nonlinear model predictive control (NMPC) strategy with a reformulated VOM for avoidance of fixed and moving obstacles. The model was evaluated through a series of simulations, which showed that the developed strategy was efficient in avoiding collisions with obstacles in complex dynamic environments and narrow spaces. Wan et al. [11] presented a deep reinforcement learning (DRL)-based method for motion control of unmanned aerial vehicles (UAVs) while flying autonomously through unknown dynamic environments providing good adaptability and navigation through them. Wang et al. [12] proposed a globally guided reinforcement learning approach (G2RL) that incorporated a novel reward structure that generalized to arbitrary environments and that, when applied to solve the multi-robot path-planning problem, proved to be robust, scalable, and generalizable and outperformed existing distributed multi-robot path-planning methods.

Shen and Zhai [13] proposed a combination of arc trajectory and fuzzy neural network approach for navigation in an unknown static environment, which produced successful obstacle avoidance for the robot, and it was stated that the approach could be applied in a dynamic environment. Wang et al. [14] created a hybrid method for path planning in a dynamic environment using a fuzzy APF to create high-quality training samples for an extensible neural network, obtaining an excellent path-planning performance in simulations and physical experiments. Furthermore, Yuan et al. [15] proposed a gated recurrent unit–recurrent neural network (GRU-RNN) dynamic path-planning model that used an improved APF and ACO algorithm for generating sample sets that provided the inputs and tags for the network. The simulation results showed the algorithm was more robust to differences in robot structure than traditional path-planning models and it provided smooth, real-time path planning. The APF method was used for emergency collision avoidance, implemented when obstacles were detected at a distance shorter than 2 m. Chen et al. [16] used an adaptive NN-based control scheme for an uncertain nonholonomic wheeled mobile robot to successfully constrain velocity and obtain a tracking error that converged to zero. Shamsfakhr and Sadeghibigham [17] implemented two feedforward NNs based on function approximation. These networks used backpropagation with a conjugate-gradient method and were trained on 3000 patterns for obstacle avoidance in an unknown, dynamic environment. The simulation experiments generated a smooth and safe path from the start point to the target position, avoiding obstacles. Inoue et al. [18] combined the rapidly exploring random tree (RRT) algorithm with a long short-term memory (LSTM) NN, and a convolutional encoder, obtaining high success for non-fluctuating path planning in previously trained environments and unknown environments similar to the trained environments. Chen et al. [19] presented a neural dynamics approach for robot collision-free path planning in dynamic environments without prior information. They corrected the traditional neural dynamic model that tends to choose a sub-optimal path when navigating by using a padding mean neural dynamic model (PMNDM) that resulted in obtaining a more efficient path generation in unknown dynamic environments.

Regarding Convolutional Neural Networks (CNNs), Medvedev et al. [20] proposed a neural-based navigation system using a trained collision avoidance D*algorithm that allowed the robot to learn from its experiences and update the parameters of a CNN, allowing the robot to navigate its environment by analyzing the situation through deep learning. The algorithm proved to be useful for supervised and reinforcement learning. Azimirad et al. [21] proposed a new approach based on a Generalized Regression Neural Network (GRNN) and optimal control. Their work first used an indirect open-loop optimal control for trajectory planning, and the neural network model was employed to reduce the time of the procedure, which trained the GRNN. With this method, path planning took an approximate time of 3.06 s. Afterwards, the optimal control was replaced by the trained GRNN for path planning, which reduced the computation time to 0.303 s, proving the superior efficiency of the trained GRNN.

Zhu et al. [22] introduced a novel approach regarding multi-robot motion planning and neural networks. They proposed a trajectory prediction model based on recurrent neural networks (RNNs) that learned multi-robot motion behaviors from demonstrated trajectories generated using a centralized sequential planner that was then implemented into a decentralized model predictive control (MPC) framework capable of multi-robot motion planning and obstacle avoidance with accurate trajectory predictions. Zheng et al. [23] proposed a laser-based people detection system based on a Deep Neural Network (DNN) using a sliding window to classify the points in a laser scan, as well as a sampling-based local trajectory planner for avoiding obstacles in a hospital environment. The experiments showed the algorithm performed successfully in real-world environments.

Yin and Yuschenko [24] proposed using a convolutional LSTM NN to achieve motion planning in one step. This was achieved by using an RGB picture with obstacles, target position, and starting position as input for the LSTM network which output the angular and linear velocity of the mobile robot and used the convolutional layer for marking obstacles, goal position, and initial position, providing an accurate path in a static environment with good fault tolerance that could be later implemented in an environment with dynamic obstacles. Molina et al. [25] used an LSTM NN with an Adam optimization for both static and dynamic obstacle avoidance in a simulated environment, obtaining a training accuracy of above 99% with the model being able to generalize untrained trajectories of under 2 m.

This work presents a physical implementation and assessment of a method for dynamic obstacle avoidance in mobile robotics using an LSTM neural network model that obtains information from the robot’s LiDAR and allows it to go from a starting point to a target point while avoiding two dynamic obstacles and multiple static obstacles. The developed algorithm is validated in a simulation and implemented in a real-life scenario using a mobile robot within a controlled motion capture environment to prove the effectiveness of the method. The implementation is carried out by adapting and integrating tools with operating limitations, such as the mobile robot, its LiDAR, and the motion capture system, to obtain a successful execution during experiments.

The rest of the paper is organized as follows: Section 2 describes artificial neural networks, ROS, and motion capture systems. Section 3 describes the experimental setup for the investigation. Section 4 presents results for the experiments, and Section 5 refers to conclusions and discussion.

## 2. Materials and Methods

### 2.1. Artificial Neural Networks

**Figure 1:**
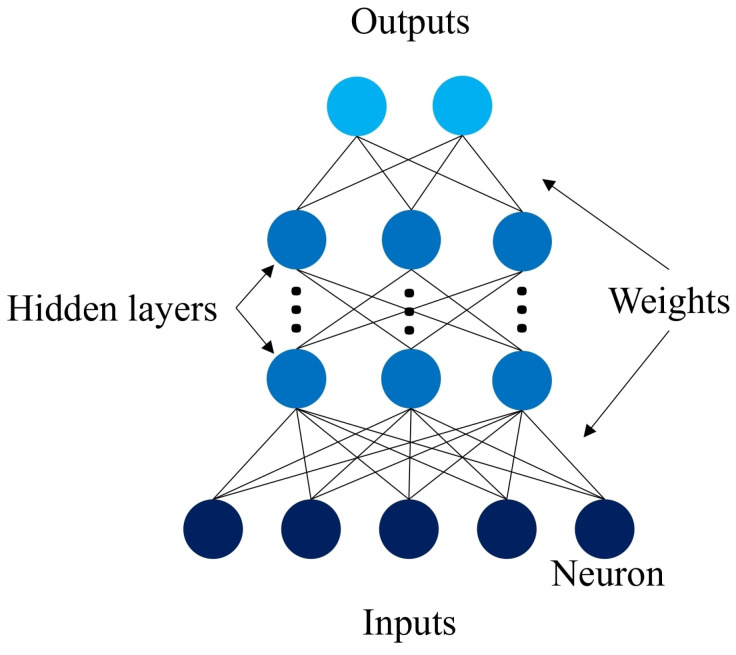
Neural network model.

Artificial neural networks (ANNs) are AI algorithms consisting of an input layer of neurons (or nodes, units), one or multiple hidden layers, and an output layer. Neurons are connected among them, with associated weights for each connection. Artificial neurons are followed by nonlinear activation functions that increase the approximation capacities beyond those of linear functions. NNs are widely used to solve the obstacle avoidance problem in dynamic environments, thanks to their ability to generalize, to learn distributed representations, and their tolerance to errors in the data [2]. A model of a simple feedforward NN is shown in Figure 1 [26].

Regarding the architecture, feedforward networks consist of a signal flow that moves in a strict forward direction from the input to the output units, with no feedback connections. On the other hand, recurrent networks do contain feedback connections with important dynamical properties. There are cases in which the activation values of the network’s units go through a relaxation process after which the network reaches a stable state, and the activation values stop changing. However, there are other cases in which the changes in the activation values are significant, so the dynamic behavior forms the network output.

There are three main learning paradigms in neural networks: supervised learning, unsupervised learning, and reinforcement learning. In supervised learning, an input vector presented is at the input layer, as well as a set of desired responses at the output layer. The discrepancies between the actual response of the system and the desired response are obtained and used to determine weight changes in the network according to the learning rule. The required information for training is obtained from an external teacher. In unsupervised learning, an output unit is trained to respond to pattern clusters within the input by statistically discovering the main features of the inputs. The system develops its representation for the inputs, so it does not have a preset group of categories in which the patterns must fall. Finally, reinforcement learning aims to maximize reward, meaning that the system discovers what actions lead to the most reward by trial-and-error [27].

#### LSTM Neural Network

The long short-term memory (LSTM) model was proposed to solve an issue with recurrent neural networks (RNNs) regarding recalling information over extended periods of time [28]. The LSTM neural network architecture consists of subnets, known as memory blocks, that are recurrently connected. These blocks contain one or more self-connected memory cells and three multiplicative units: the input, output, and forget gates. These provide continuous analogues of read, write, and reset operations for the cells. The multiplicative gates allow the memory cells from the LSTM model to store and access information over long periods. This architecture has helped greatly over the past years to solve multiple artificial problems that had proven impossible to solve using regular recurrent neural networks [29]. The following equations describe the LSTM network’s units [30]:

(1) $$f_{t}=\sigma \left(W_{f}\left[h_{t-1},x_{t}\right]+b_{f}\right)$$

(2) $$i_{t}=\sigma \left(W_{i}\left[h_{t-1},x_{t}\right]+b_{i}\right)$$

(3) $${\tilde{C}}_{t}=tanh\left(W_{C}\left[h_{C-1},x_{t}\right]+b_{C}\right)$$

(4) $$C_{t}=f_{t}⊙C_{t-1}+i_{t}⊙{\tilde{C}}_{t}$$

(5) $$o_{t}=\sigma \left(W_{o}\left[h_{t-1},x_{t}\right]+b_{o}\right)$$

(6) $$h_{t}=o_{t}⊙tanh\left(C_{t}\right)$$

**Figure 2:**
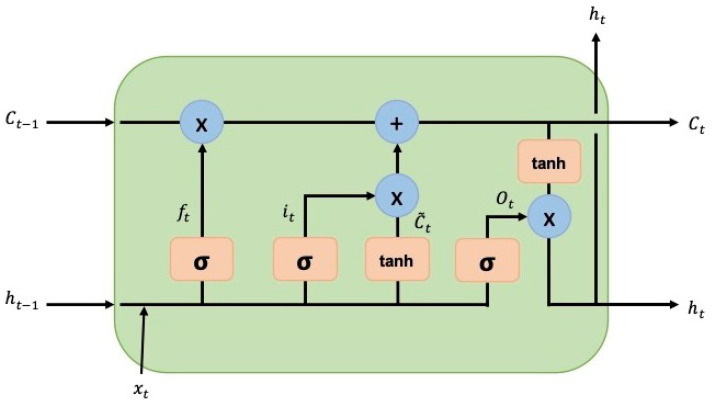
LSTM cell block diagram.

In the equations shown above, $f_{t}$, $i_{t}$, and $o_{t}$ are the forget, input, and output gate vectors, respectively. σ is a nonlinear function, $W_{f}$, $W_{i}$, $W_{o}$, and $W_{C}$ are weight matrices, $h_{t-1}$ is the hidden value at the previous timestamp, $x_{t}$ is the input to the memory cell, $b_{f}$, $b_{i}$, $b_{o}$, and $b_{C}$ are bias vectors, ${\tilde{C}}_{t}$ is the new cell content, $C_{t}$ is the cell activation vector, and $h_{t}$ is the hidden value. The use of memory cells, instead of recurrent units, helps ease the long-timescale learning of temporal relationships [31]. Figure 2 shows an LSTM cell block [30].

### 2.2. Robot Operating System (ROS)

The Robot Operating System (ROS) is a set of libraries and tools to develop software for robots that has an emphasis on large-scale integrative robotics research [32]. It is considered the de facto standard for robot programming due to the thousands of available packages. It is an open-source project that offers a core set of software for operating robots that can be extended by using existing packages or creating new ones, making it possible to develop robotic software that can be reused on different hardware platforms. It relies on contributions from developers worldwide, and its applications communicate using a publisher–subscriber architecture, allowing users to build software that includes sophisticated functionality such as path planning, object recognition, and grasping [33].

ROS has five main characteristics. The first one is that it uses peer-to-peer connectivity along with buffering to avoid creating traffic in the wireless link between hosts. It is also multi-lingual, allowing users to write code in several different languages, such as Python, C++, Lisp, and others in development. Furthermore, the software is tool-based, meaning it uses multiple small tools that perform numerous tasks to create and run its different components, and it enforces algorithm development that has no dependencies on ROS, resulting in standalone libraries that can be reused for multiple projects. Finally, it is free and open-source.

The fundamental concepts to understand for using ROS are nodes, topics, messages, and services. Nodes are computing processes, and a ROS system typically consists of multiple nodes. These nodes need to communicate with each other, and they do so by passing messages, which are data structures with rigorous types. The messages are sent between nodes by being published to a certain topic, defined as a string to which the nodes can subscribe if they are interested in its specific data type. Finally, services are used for synchronous transactions. They are defined using a string name and two messages: one for the request and the other one for the response [32].

### 2.3. Motion Capture Systems

Motion capture (MoCap) systems digitally track and record the movements of living beings and objects in space. Over the years, there have been different technologies developed to capture motion. Camera-based optoelectronic systems use infrared cameras that triangulate the location of retroreflective rigid bodies attached to the target object [34]. These systems are based on fixed cameras, meaning that they can acquire data in a specific, restricted area. There are two categories for this type of system: active marker systems and passive marker systems. Active marker systems use markers with their own source of light for the sensors, while passive marker systems use markers that reflect the light back to the sensors [35].

Other systems use depth-sensitive cameras, which project light toward a target object and estimate the depth based on the time delay from the light emission to backscattered light detection [34]. There are systems based on other technologies, such as image processing, electromagnetic measurement, ultrasonic localization, and inertial sensor measurement, among others [35], and there are hybrid systems, which combine MoCap technologies to improve location precision.

MoCap technologies are employed in numerous applications, such as healthcare, clinical settings, sports, entertainment, the gaming industry, robotics, automotive, and construction. In healthcare, these systems aid in the diagnosis and treatment of physical conditions by studying a patient’s motor function or by comparing recordings to evaluate the effect of the rehabilitation approach. In sports, MoCap systems can study an athlete’s motion to analyze how efficient their athletic posture is and make recommendations that could enhance their performance. Regarding industrial settings, MoCap is broadly used in industries such as gaming and entertainment, with a few implementations in industries such as robotics, automotive, and construction. These sectors, however, require highly specialized equipment, regular and precise calibration routines, high capture volumes, and technical suits, which have impeded implementing this system in primary and secondary industrial applications [34]. In the present work, a camera-based optoelectronic MoCap setup with a passive marker system was used to obtain the position of the mobile robot at each time instance.

## 3. Experimental Setup

### 3.1. TurtleBot3 Waffle Pi

The robotic platform on which the implementation was tested was the TurtleBot3 Waffle Pi by ROBOTIS (ROBOTIS Co., Ltd. 37, Magokjungang 5-ro 1-gil, Gangseo-gu, Seoul, Republic of Korea). It is a ROS-based, programmable robot that can be customized in various ways depending on the assembly of its mechanical parts. The robot is equipped with a 2D LiDAR LDS-02 sensor, a Raspberry Pi4 single-board computer (Raspberry Pi Foundation, Hills Road, Cambridge, England, UK), a 32-bit ARM Cortex^®^-M7 with FPU (216 MHz, 462 DMIPS) MCU (ARM, 110 Fulbourn Road Cambridge, UK), an OpenCR 1.0 board, two DYNAMIXEL XM430-W210-T motors, and a Raspberry Pi Camera Module v2.1 (Raspberry Pi Foundation, Hills Road, Cambridge, England, UK). It has a maximum translational velocity of 0.26 m/s and a maximum rotational velocity of 1.82 rad/s. The robot was assembled, and its components were connected following the ROBOTIS e-Manual for the TurtleBot3 Waffle Pi. The setups for both the Raspberry Pi and the OpenCR board were also established following the e-manual.

**Figure 3:**
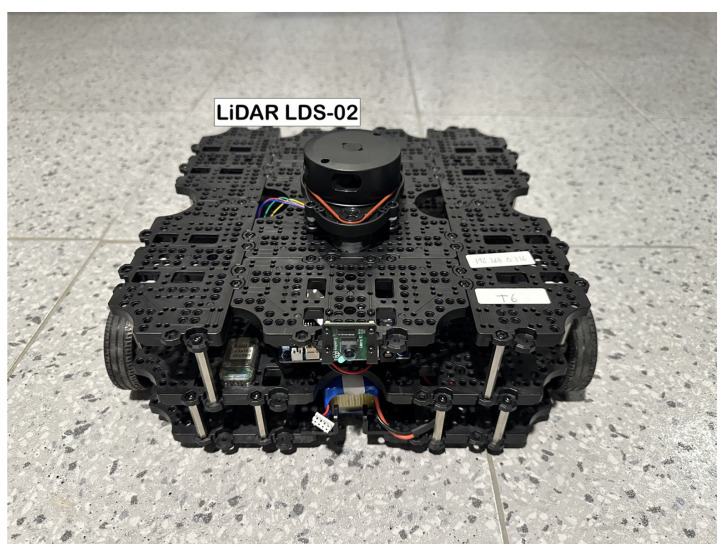
TurtleBot3 Waffle Pi with LiDAR sensor.

The 2D LiDAR LDS-02 sensor in the robot is a 360-degree laser distance sensor used to perceive the environment. It emits a laser and measures its intensity and flight time after being reflected from the object to obtain the distance at which it is located. It has a sampling rate of 2.3 kHz, an operating range from 160 mm to 8000 mm, and an angular resolution of 1°, which can vary depending on the scan frequency of the LiDAR. The robot and its LDS-02 LiDAR sensor are shown in Figure 3.

**Figure 4:**
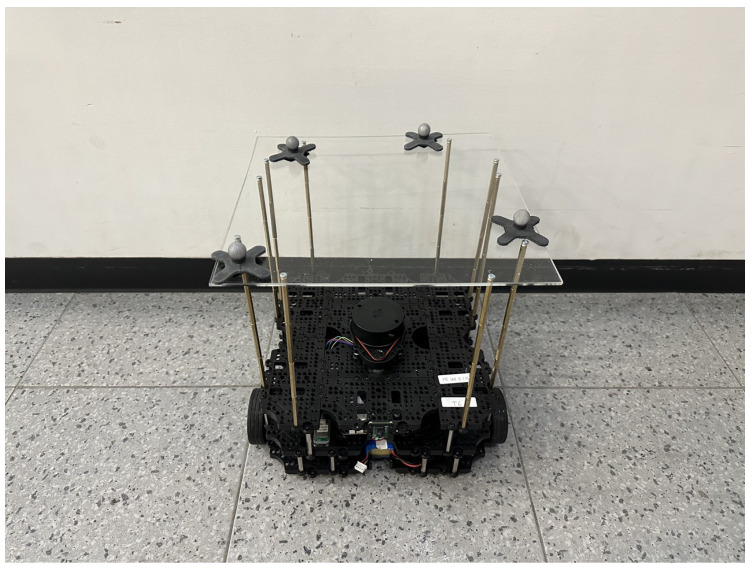
Placement of four retroreflective markers on a TurtleBot3 for obtaining the robot’s position in the MoCap system.

For the MoCap system to be able to provide the location of the robot in the area, four retroreflective markers were attached to the robot. These markers reflected light to the cameras in the MoCap system so their location could be triangulated and shown in the software. The markers had to be at a height of 30 cm and positioned in an asymmetrical pattern for the system to work accurately when calculating the position of the rigid body; thus, an extra platform was attached to the robot for the markers to be at the desired height. Figure 4 shows the chosen placement of the markers on the TurtleBot3.

### 3.2. OptiTrack-Motive Motion Capture System

**Figure 5:**
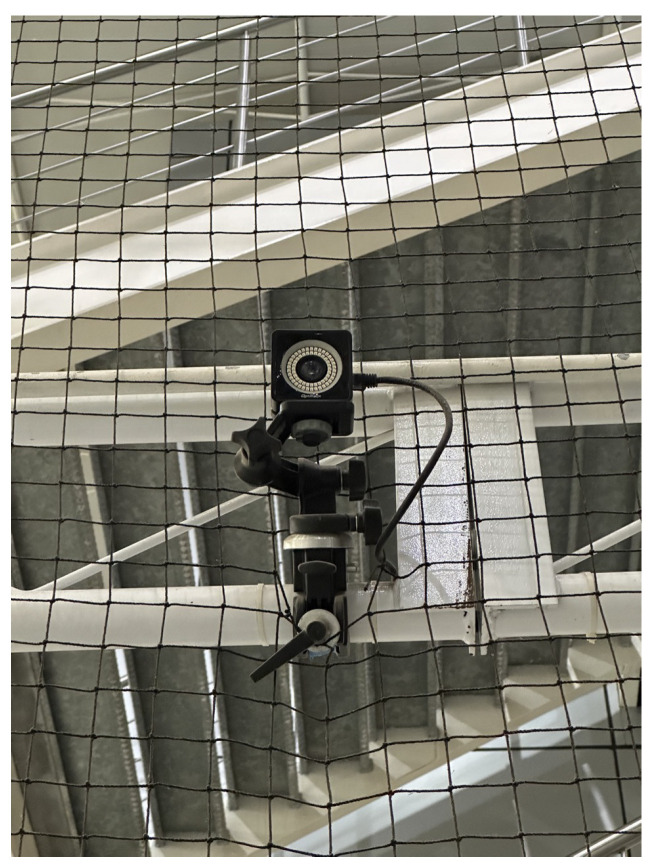
OptiTrack Prime 13 camera from the OptiTrack-Motive Motion Capture system.

The physical implementation occurred in the OptiTrack-Motive motion capture System at Tecnológico de Monterrey Campus Querétaro. It is a camera-based optoelectronic MoCap setup with a passive marker system. It consists of a space measuring 8 m × 8 m × 8 m, enclosed by a net. It contains 16 OptiTrack Prime 13 MoCap cameras by NaturalPoint Inc. (NaturalPoint, 3658 SW Deschutes St, Corvallis, OR, USA), shown in Figure 5, encircling the space. Eight of the cameras are located at a height of 3 m from the ground and the other eight cameras are at a height of 8 m. The cameras are calibrated and referenced to a point in the center of the arena at a height of 3 m.

**Figure 6:**
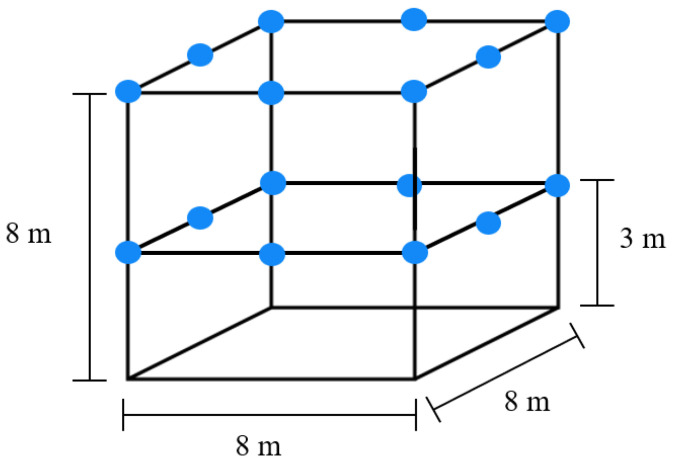
OptiTrack-Motive motion capture system model.

**Figure 7:**
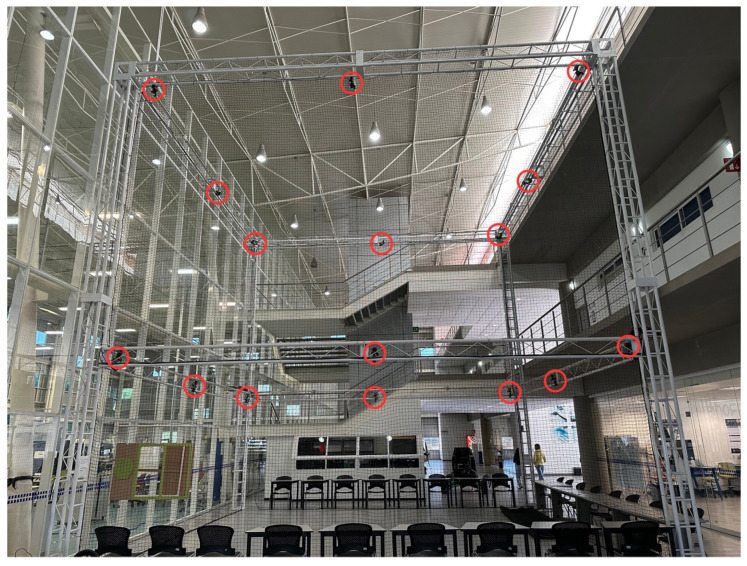
Optitrack-Motive motion capture system with the cameras highlighted by red circles.

A simple illustration of the system is shown in Figure 6, where the blue circles represent the position of each of the 16 cameras. A picture of the MoCap system is shown in Figure 7, where the cameras are marked with red circles. The cameras provide accurate positioning of markers in a 3D plane through the Motive 2.2 software, which was used in the implementation for obtaining the robot’s position. Since the robots were located at near-ground height, only the data from the 8 lower cameras was used.

The data obtained from the Motive 2.2 software were streamed using a wireless network and migrated to ROS Noetic through a ROS driver developed by Aarsh [36], which is compatible with the NatNet 4.0 protocol used by the Motive software. With these data and the data obtained from the 2D LDS-02 LiDAR sensor, the LSTM model’s performance was tested in the physical environment using the TurtleBot3 Waffle Pi.

**Figure 8:**
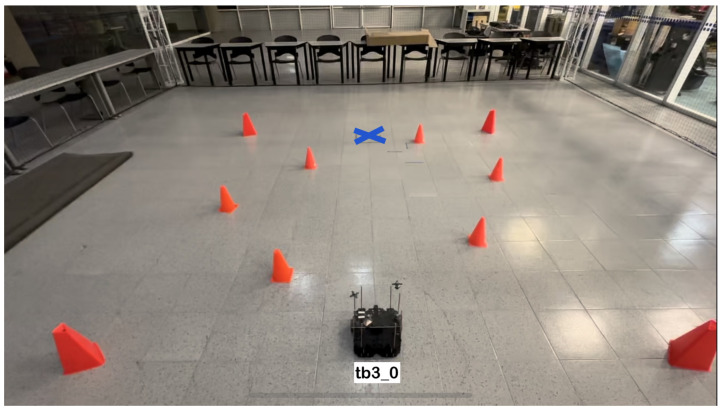
First training scenario. The starting point for the mobile robot is (−0.3, −1.2) and the goal point (−0.3, 1.8) is represented with a blue X mark. There is no dynamic obstacle. The robot must follow a straight-line path to reach its target.

**Figure 9:**
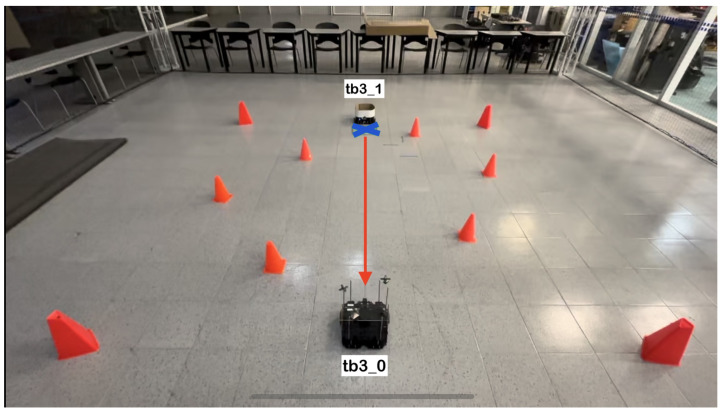
Second training scenario. The starting point for the mobile robot is (−0.3, −1.2) and the goal point (−0.3, 1.8) is represented with a blue X mark. The dynamic obstacle starts at (−0.3, 2.1) and follows a straight path toward the robot, shown with a red arrow. The robot must avoid the dynamic obstacle by deviating slightly to the left, returning to its course once the obstacle is no longer in it path.

**Figure 10:**
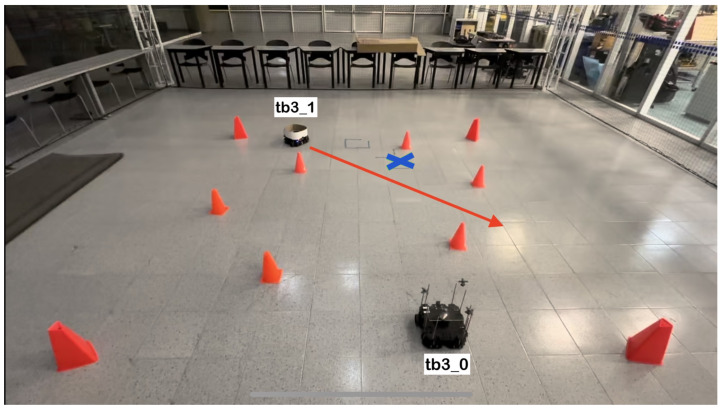
Third training scenario. The starting point for the mobile robot is (0.2, −1.2) and the goal point (0.2, 1.2) is represented with a blue X mark. The dynamic obstacle starts at (−1.0, 1.8) and follows a diagonal path crossing in front of the robot, shown with a red arrow. The robot must advance, stop to let the obstacle cross in front of it, and continue its path once there is no risk of collision.

**Figure 11:**
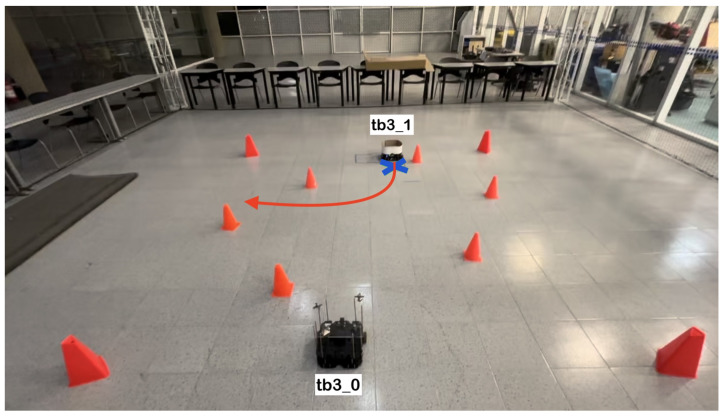
Fourth training scenario. The starting point for the mobile robot is (−0.4, −1.2) and the goal point (0.0, 1.5) is represented with a blue X mark. The dynamic obstacle starts at (0.0, 1.8) and follows a curved path that crosses in front of the robot, shown with a red arrow. The robot must go straight, deviate to the right when the obstacle changes direction, and continue its course to the target point.

**Figure 12:**
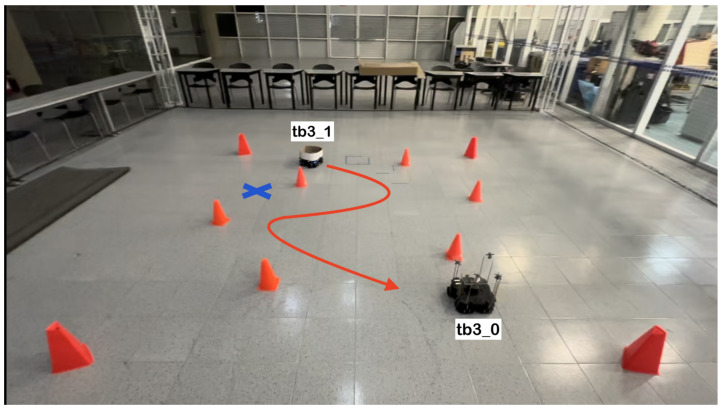
Fifth training scenario. The starting point for the mobile robot is (0.5, −0.9) and the goal point (−1.5, 0.8) is represented with a blue X mark. The dynamic obstacle starts at (−0.9, 1.6) and follows a curved, zigzag-like path, shown with a red arrow. The robot must turn 45° to the left, follow a straight path, stop to let the obstacle cross in front of it and continue its course to the goal.

The physical setup inside the MoCap system consisted of six static obstacles simulating a narrow aisle and a dynamic obstacle represented by a TurtleBot3 Waffle Pi with different starting points depending on the training scenario. Five different navigation scenarios for the mobile robot were implemented. These scenarios are shown in Figure 8, Figure 9, Figure 10, Figure 11 and Figure 12, with tb3_0 being the mobile robot and tb3_1 being the dynamic obstacle. All static obstacles remained fixed throughout the five scenarios.

### 3.3. Software Required for the Implementation

Multiple software tools were required for the implementation of this project. Most of these were run on a Dell G15 5520 computer (Dell Technologies, 1 Dell Way, Round Rock, TX, USA) with an Intel Core i7 12700H processor (Intel, 2200 Mission College Blvd, Santa Clara, CA, USA), 16 GB RAM, and an Nvidia GeForce RTX 3060 graphics card (Nvidia, 2788 San Tomas Expy, Santa Clara, CA, USA). The experiments were conducted using the Ubuntu 20.04 (Ubuntu, 5 Blue Fin Bldg, 110 Southwark St, LDN, UK) Linux distribution.

Using Ubuntu 20.04, ROS Noetic was installed, along with the required packages. It was also necessary to install Python 3.8.10 for writing scripts to control the dynamic obstacle, write to a .csv file, and train, validate, and test the neural network. Keras 2.13.1 was also installed for modeling the LSTM network.

**Figure 13:**
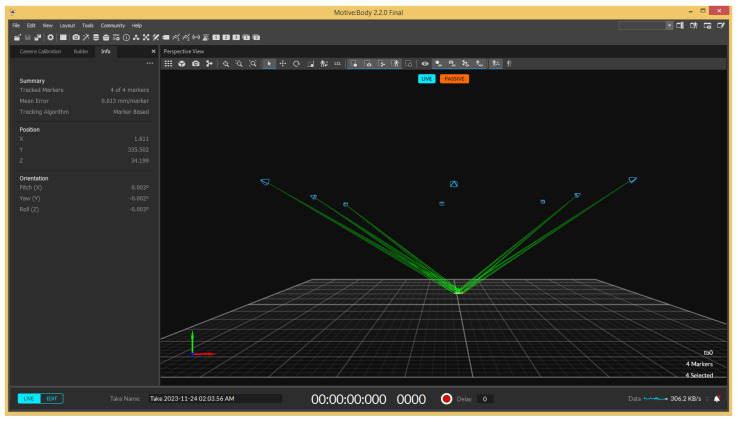
Motive 2.2’s interface showing the robot as a rigid body.

An extra computer running the Motive 2.2 software for the MoCap system was used. Motive paired with the OptiTrack cameras in the physical setup to track the motion of retroreflective markers attached to the robot, providing real-time position data with high accuracy. An example of how the software’s interface looks like is shown in Figure 13.

**Figure 14:**
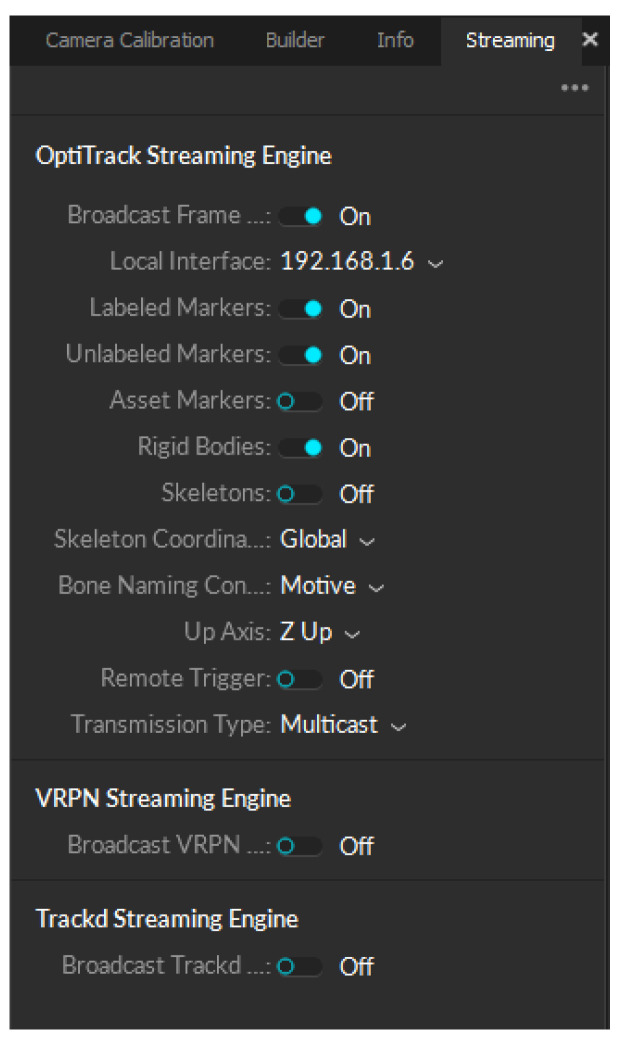
Streaming settings in Motive for migrating data using the NatNet protocol.

The data obtained from the software were migrated to ROS using the NatNet protocol, which streamed live MoCap data across a network shared between the streaming and receiver computers so it could be used for training the neural network. Certain settings had to be modified in the streaming pane of the Motive software for the protocol to work correctly. These settings are shown in Figure 14.

### 3.4. Robot Data Acquisition

The data used for the neural network’s training consisted of the LiDAR sensor’s readings, the target point for the robot, its linear and angular velocities, and its position given by the MoCap system every 0.2 s. The LiDAR readings recorded were those taken every two degrees, spanning the front 180° of the robot. Due to the varying sampling rate of the physical LiDAR, the values were filtered using a code that obtained the laser’s readings, organized them into an array containing the 90 readings from the front of the robot, and published them to a new topic. Since the specifications for the LiDAR stated that its detection distance range was 0.16–8 m, any reading greater than 8 m was replaced by the maximum value. A ROS node subscribed to the new laser scan topic of the TurtleBot3 LiDAR scan data, the linear and angular velocities of the robot given by the “cmd_vel” topic at each time instance, and the robot’s position given by the MoCap system.

The robot’s position at each time instance was obtained through the Motive 2.2 software. Four retroreflective markers were positioned on the robot in an asymmetric pattern so the cameras could track its position accurately. The position on the *x* and *y* axis obtained by the OptiTrack MoCap system was streamed to Motive using the NatNet protocol driver and the data were migrated to ROS through a wireless network, which was then recorded to a .csv file, along with the 90 laser readings, the target point for the robot, and its linear and angular velocities, for training the LSTM model. The robot was operated by the user 20 times in each of the five training scenarios, yielding a total of 100 trajectories for training the LSTM model.

### 3.5. LSTM Neural Network Implementation

**Figure 15:**
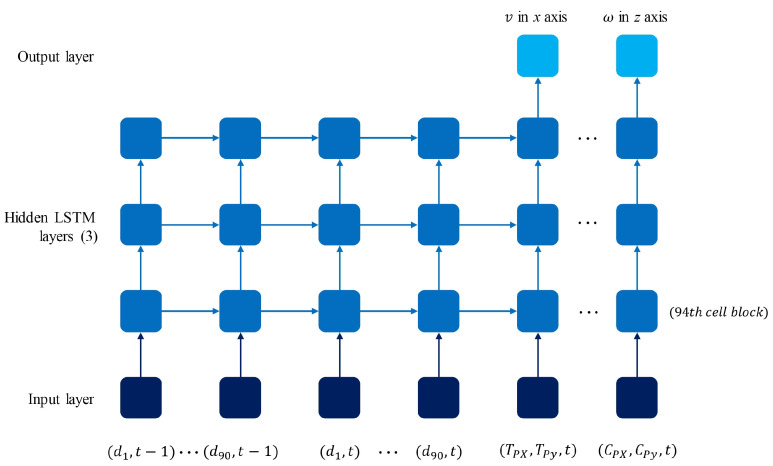
LSTM model architecture with data inputs and outputs.

The architecture of the implemented LSTM neural network, Figure 15, consisted of an input layer of 188 blocks that contained 94 features describing the position of the robot ($C_{Px},C_{Py}$), the target location ($T_{Px},T_{Py}$), and the data from the laser scan ($d_{1}-d_{90}$), all at timestep *t*, as well as the data from the laser scan at timestep t−1. Next, it contained three hidden layers of 94 LSTM cell blocks each. The first two layers were set to return a sequence output, meaning they provided an output for each timestep to the layer below. The output layer contained two neurons that returned the linear and angular velocity of the mobile robot in *x* and *z*, respectively. The user-recorded data in the .csv file were reshaped using the Keras library to fit the batch size, timesteps, and features parameters of the LSTM model, considering the features and the previous timestep. The loss of the network was calculated as the Mean Squared Error (MSE). Once the data were ready, the model was trained for 400 epochs with a batch size of 32, using an Adam optimization.

Since the model dealt with multiple trainable parameters, dropout was used to prevent overfitting with 30% dropout layers added between the hidden layers. A ROS node subscribed to the laser scan topic and obtained online LiDAR sensor data, publishing to the velocity topic with the LSTM-predicted velocities for the mobile robot. The roslaunch tool started three ROS nodes: one with the obstacle controller algorithm, one with the LSTM model testing navigation, and a third one that obtained the MoCap system’s data regarding the mobile robot’s position so that the navigation of the robot with the proposed model could be tested in the designated area.

## 4. Results

The model was trained using 100 different trajectory examples through five different scenarios, consisting of an instance where there was no dynamic obstacle and another four scenarios with different starting and target points for the mobile robot, as well as different trajectories for the dynamic obstacle in each of them. The first two scenarios analyzed what the robot would do when moving through a narrow aisle with no obstacles versus when encountering a dynamic obstacle directly in front of it. The other three scenarios studied the robot’s behavior when the dynamic obstacle presented more complex trajectories, such as crossing its path diagonally, changing directions suddenly, or following a more complex, curved, zigzag-like path that crossed the path of the robot toward its goal.

**Figure 16:**
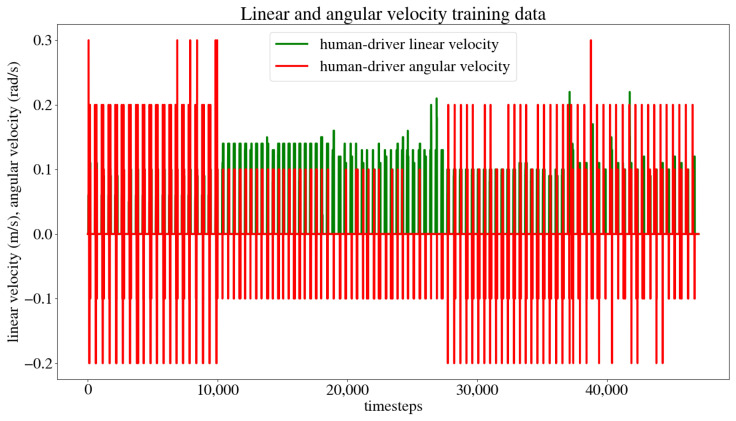
Linear and angular velocity commands given by the user during training.

The criteria used for determining whether the model was successful in its task were as follows: the mobile robot had to reach its target within a 10 cm radius of the original goal point, and it had to do so while avoiding collisions with both static and dynamic obstacles. The time it took for the robot to reach its target was also recorded. Each scenario was run through twenty times to ensure the model had enough data for the robot to move autonomously when testing the LSTM model. Figure 16 shows the linear and angular velocity commands given by the user during all the training instances for the five cases. The *x* axis, “timesteps”, represents each instance the data were recorded, which was every 0.2 s. Therefore, one timestep = 0.2 s. The number of timesteps required for the robot to reach its goal in each scenario depended on the proximity of the target point and the complexity of the path it needed to follow.

**Figure 17:**
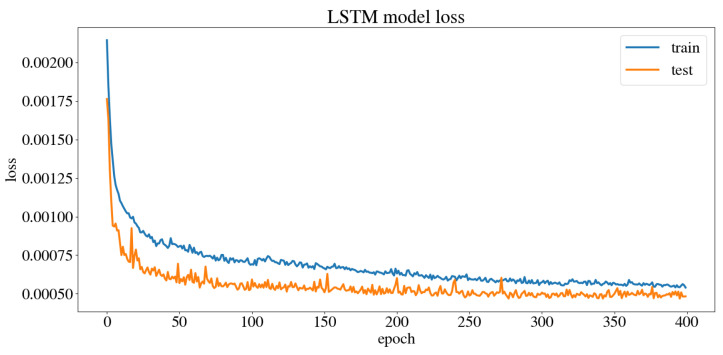
Model training and test loss curves.

**Figure 18:**
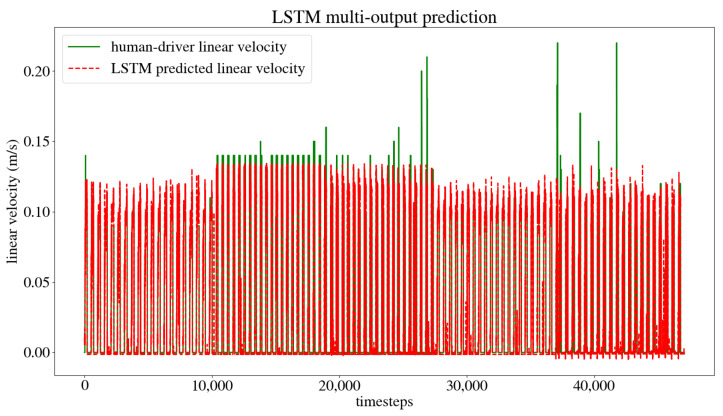
Linear user-trained versus model-predicted velocities.

**Figure 19:**
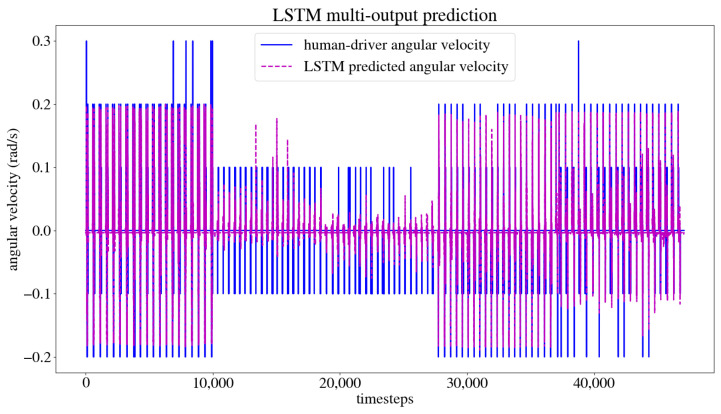
Angular user-trained versus model-predicted velocities.

The dataset obtained from the user’s navigation of the five trajectories was split randomly into 80% for the training set and 20% for the test set. The model was trained and validated, obtaining the model training and test loss curves presented in Figure 17, which show that the model reaches a minimum loss throughout the training epochs without overfitting or underfitting. After training, an MSE of 0.0003 m/s was obtained for the linear velocity in the *x* axis, as well as an MSE of 0.0007 rad/s for the angular velocity in the *z* axis, and an accuracy of 98.02% when evaluated by the model. This accuracy is calculated by dividing the number of output values that the model predicted correctly by the total number of output values, obtaining the percentage of accurately predicted values. Figure 18 compares the linear velocities given during training versus the ones predicted by the model, and Figure 19 does the same with the angular velocities.

**Figure 20:**
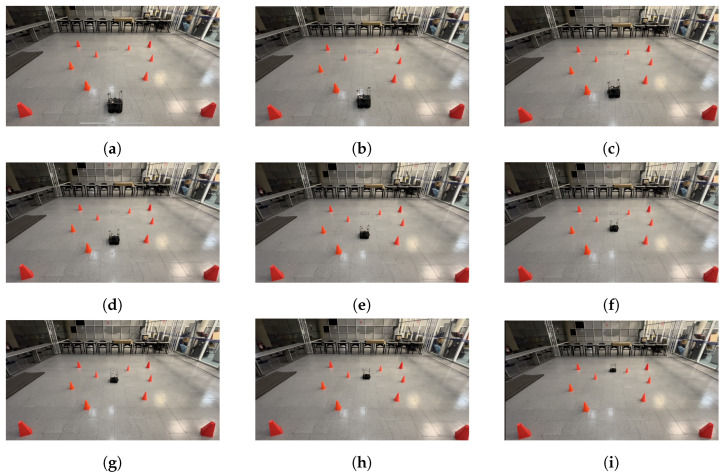
Scenario 1: The target point for the robot is (−0.3, 1.8). There is no dynamic obstacle. (**a**) shows the starting point of the robot, (**b**–**h**) show the trajectory that it followed, and (**i**) shows the robot at its goal.

**Figure 21:**
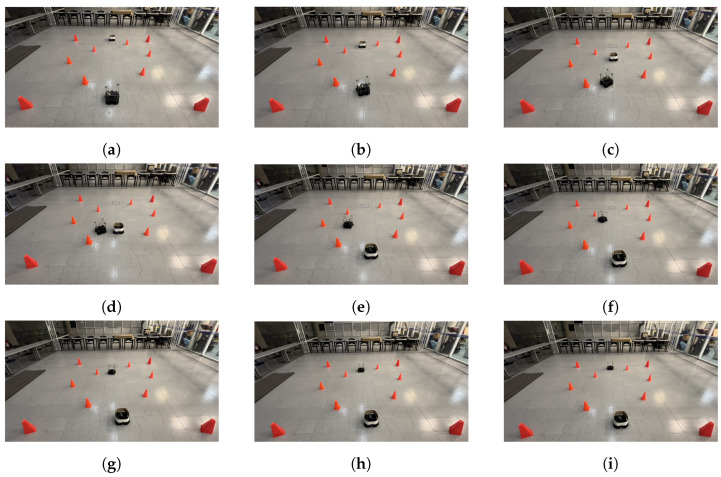
Scenario 2: The target point for the robot is (−0.3, 1.8). The dynamic obstacle travels in a straight line toward the robot. (**a**) shows the starting point of the mobile robot and dynamic obstacle, (**b**–**h**) show the trajectory followed by both and (**i**) shows the mobile robot at its goal, as well as the dynamic obstacle at its ending point.

**Figure 22:**
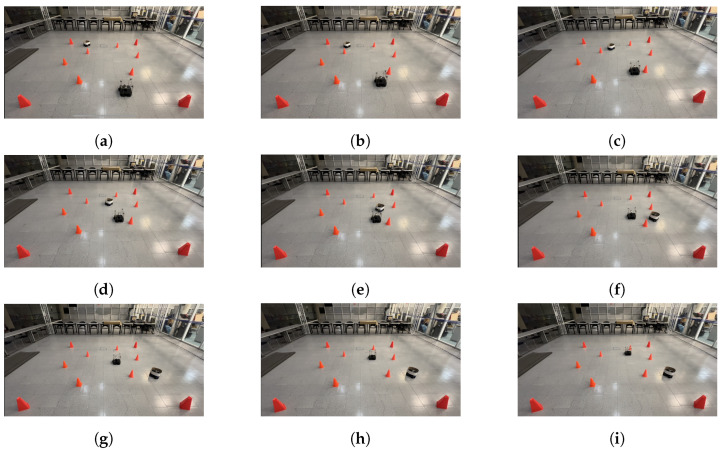
Scenario 3: The target point for the robot is (0.2, 1.2). The dynamic obstacle crosses the robot’s path in a diagonal trajectory. (**a**) shows the starting point of the mobile robot and dynamic obstacle, (**b**–**h**) present the trajectory followed by both, and (**i**) shows the mobile robot at its goal, as well as the dynamic obstacle at its ending point.

The LSTM model was tested in the same five scenarios. In the first scenario, the starting point for the robot was (−0.3, −1.2) and its target point was (−0.3, 1.8). There was no dynamic obstacle, so the robot moved straight ahead towards the target point. The trajectory followed by the robot is shown in Figure 20. In the second scenario, the starting and target points for the mobile robot were still (−0.3, −1.2) and (−0.3, 1.8), respectively. However, the dynamic obstacle was placed in front of the robot at (−0.3, 2.1) and followed a linear trajectory, so the robot took a small curve to avoid the obstacle and get to the target point, as seen in Figure 21. In the third scenario, the robot was initially placed at (0.2, −1.2), and its target point was (0.2, 1.2). The starting point for the dynamic obstacle was (−1.0, 1.8), and it crossed the robot’s path in a diagonal trajectory, forcing the robot to stop and let the obstacle through, continuing its path afterward. The interaction is shown in Figure 22.

**Figure 23:**
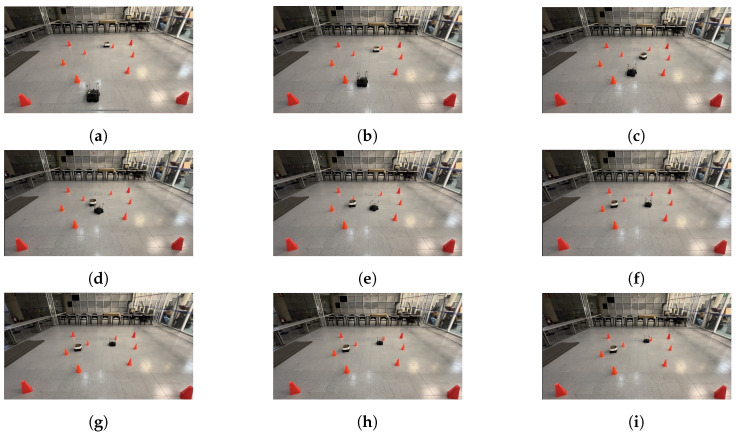
Scenario 4: The target point for the robot is (0.0, 1.5). The dynamic obstacle follows a curved trajectory that crosses the robot’s path. (**a**) shows the starting point of the mobile robot and dynamic obstacle, (**b**–**h**) show the trajectory followed by both, and (**i**) shows the mobile robot at its goal, as well as the dynamic obstacle at its ending point.

**Figure 24:**
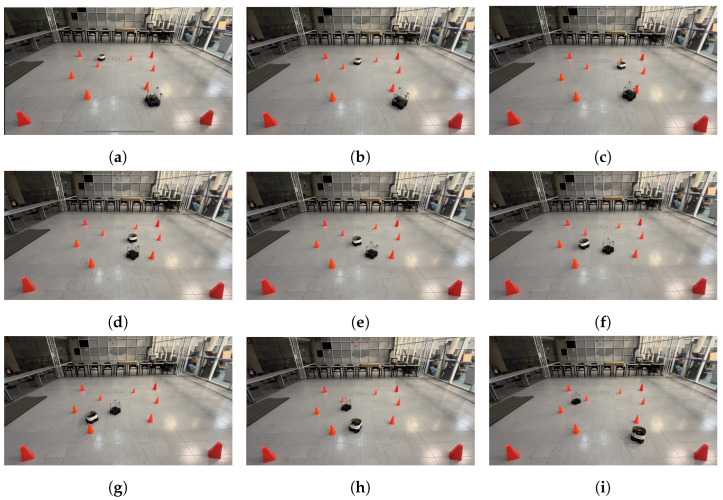
Scenario 5: The target point for the robot is (−1.5, 0.8). The dynamic obstacle follows a curved zig-zag trajectory, crossing the robot’s path. (**a**) shows the starting point of the mobile robot and dynamic obstacle, (**b**–**h**) show the trajectory followed by both and (**i**) shows the mobile robot at its goal, as well as the dynamic obstacle at its ending point.

Figure 23 illustrates the fourth scenario, in which the robot started its trajectory at (0.4, −1.2), and its target point was (0.0, 1.5), the dynamic obstacle was placed in front of the robot, in the position (0.0, 1.8), and it followed a curved “L” trajectory, forcing the robot to avoid it. Finally, Figure 24 shows the fifth scenario. Here, the starting point for the robot was (0.5, −0.9) with the target point being (−1.5, 0.8). The dynamic obstacle was placed at (−0.9, 1.6) and it followed a curved zig-zag-like trajectory that crossed the robot’s path and forced it to stop and let the obstacle pass, continuing its course afterward.

The model was tested multiple times in the same circumstances to ensure the robot reached the desired position while avoiding dynamic and static obstacles in the different scenarios. Each time, the mobile robot reached its target within a 10 cm radius from the original goal point, and there were no collisions with either static or dynamic obstacles in all scenarios. The results for the time it took the robot to reach its goal in each case are presented in Table 1.

As seen in Table 1, the presented model achieved its purpose while meeting the previously set criteria for success, in 40 s or less in each case. Through numerous runs of the five scenarios in which the mobile robot had to avoid static and dynamic obstacles to reach a goal point, it did so every time it was tested. The target was reached with an error of less than 10 cm while avoiding collisions with the dynamic and static obstacles, even in situations where the mobile robot had to stop, let the dynamic obstacle through, and continue its path afterward, proving the effectiveness of the proposed method.

## 5. Conclusions

An approach to dynamic obstacle avoidance based on an LSTM neural network was implemented using two TurtleBot3 robots to represent the navigating robot and a dynamic obstacle. The robot navigated through its environment using LiDAR readings taken every 2° from the front of the robot in a range from 0° to 180°, and its position was obtained using a MoCap system. The robot was given 100 navigating examples through five different scenarios, which were recorded in a .csv file that included the 90 readings from the robot’s LiDAR, the target point, the robot’s position on the *x* and *y* axis obtained from the MoCap system, and the robot’s linear and angular velocities at each time instance. The LSTM network was trained using these data. The training results showed an accuracy of 98.02% for the model’s trajectory prediction, as well as an MSE of 0.0003 m/s for the linear velocity along the *x* axis, and an MSE of 0.0007 rad/s for the angular velocity along the *z* axis.

The model was tested multiple times, studying its ability to reach the desired goal in each of the five scenarios, with different and complex trajectories for the dynamic obstacle. In each experiment, the robot reached the target point while avoiding collision with the dynamic obstacle, either by changing the angle of its trajectory or by stopping, letting the dynamic obstacle through, and then continuing on its path. The physical implementation of an LSTM neural network model proved to be successful in allowing a robot to navigate from a starting point to a target point while avoiding a dynamic obstacle in five different scenarios.

Future work with the model includes evaluating the algorithm against popular dynamic obstacle avoidance algorithms such as DWA, APF, and VMO, implementing the model in a less controlled scenario, and using robotic vision for obtaining the robot’s position in different time instances instead of the MoCap system. Other scenarios could also be explored, such as having the velocity of the dynamic obstacle vary, implementing multiple dynamic obstacles, and adding more training scenarios so the model is more robust. 

## Figures and Tables

**Table 1:** Experimental results from testing the model on five different situations.

Scenario	Time
Scenario 1	
Goal point (−0.3, 1.8)	27 s
Scenario 2	
Goal point (−0.3, 1.8)	38 s
Scenario 3	
Goal point (0.2, 1.2)	28 s
Scenario 4	
Goal point (0.0, 1.5)	34 s
Scenario 5	
Goal point (−1.5, 0.8)	40 s

## Data Availability

The data necessary for the reproduction of the experiments can be found in the GitHub repository https://github.com/esmemt/drone_arena (accessed on 8 May 2024).
